# Exclusion of large herbivores affects understorey shrub vegetation more than herb vegetation across 147 forest sites in three German regions

**DOI:** 10.1371/journal.pone.0218741

**Published:** 2019-07-10

**Authors:** Deborah Schäfer, Daniel Prati, Peter Schall, Christian Ammer, Markus Fischer

**Affiliations:** 1 Institute of Plant Sciences, University of Bern, Bern, Switzerland; 2 Silviculture and Forest Ecology of the Temperate Zones, University of Göttingen, Göttingen, Germany; Helmholtz Zentrum Munchen Deutsches Forschungszentrum fur Umwelt und Gesundheit, GERMANY

## Abstract

**Background:**

Many studies have analysed the effect of browsing by large herbivores on tree species but far fewer studies have studied their effect on understorey shrubs and herbs. Moreover, while many studies have shown that forest features and management intensity strongly influence understorey vegetation, the influence of such variation on the effect of large-herbivore exclusion is not known.

**This study:**

In this study, we analysed changes of species richness, Shannon diversity, evenness and cover of understorey herbs and shrubs after excluding large herbivores for seven years on 147 forest sites, differing in management intensity and forest features, in three regions of Germany (Schwäbische Alb, Hainich-Dün, Schorfheide-Chorin). Further, we studied how the effect of large-herbivore exclusion on understorey vegetation was influenced by forest management intensity and several forest features.

**Results:**

As expected, exclusion of large herbivores resulted in highly variable results. Nevertheless, we found that large-herbivore exclusion significantly increased cover and Shannon diversity of shrub communities, while it did not affect herb communities. Forest management intensity did not influence the effect of large-herbivore exclusion while some forest features, most often relative conifer cover, did. In forests with high relative conifer cover, large-herbivore exclusion decreased species richness and cover of herbs and increased Shannon diversity of herbs and shrubs, while in forests with low relative conifer cover large-herbivore exclusion increased species richness and cover of herbs, and decreased Shannon diversity of herbs and shrubs.

**Conclusion:**

We suggest that browsing by large herbivores should be included when studying understorey shrub communities, however when studying understorey herb communities the effects of browsing are less general and depend on forest features.

## Introduction

The influence of herbivores on the diversity and composition of Central European forest communities is subject to a long and controversial debate (e.g. [1,2]). Humans have driven many large predators to extinction and hunting does not seem to fully compensate the lack of predators [3,4]. Together with the reduction of natural predators and partly low hunting intensity, an increase in forage quality through agricultural and silvicultural activities (i.e. overall higher nutrient input) and landscape fragmentation has led to a higher number of large herbivores during the last decades [5–7]. These large herbivores, in Central Europe mostly red deer, roe deer, fallow dear and wild boars, can severely damage forest plants by consuming herbs, buds of shrubs and trees, and plant roots. However, the extensive literature on effects of large herbivores in forests mostly focused on tree species [6,8,9], including studies on tree seedlings and saplings [10,11], showing reduced growth [12], shifts in size structure [13,14] and most importantly a homogenisation of tree composition [15,16]. In contrast, the influence of large herbivores on understorey shrubs and herbs has been less studied, even though understorey vegetation is important for abundance and species richness of other organisms, forest succession and ecosystem processes [6,17–20]. Studies that did analyse the influence of large herbivores on understorey vegetation found for example that large herbivores may affect plant cover, species richness or the homogenization of the understorey vegetation (i.e. increase in dominance of some species) [5,9,21–25]. However, previous studies were either not located in Central Europe [25], focused on a single type of forest (e.g. broadleaf forests [21]; ancient forests, [22]) or were constrained in their number of replicates and therefore struggled with the large variation of treatment effects [6,22,23]. Thus, for a comprehensive assessment of the role of herbivory in Central European forests, more multi-site and large-scale studies across different forest types are needed that include shrub and herb species in understorey vegetation.

For several reasons the effect of large-herbivore exclusion on understorey plant communities is expected to be highly variable. Large herbivores differ in their preference for different forest types [26] and plant species [24,27–29], thereby showing pronounced site- and species-specific effects. Whereas the effect of large herbivores is generally considered a disturbance for plants, due to the damage caused while browsing, plants differ in their sensitivity to browsing by herbivores. Some species can tolerate damage by large herbivores [6,13,30] or even overcompensate moderate damage caused by herbivores [6,13,29], thus sometimes shifting entire forest plant communities [6,19]. In addition, large herbivores can affect understorey vegetation positively, for instance by promoting seed dispersal by endo- or epizoochory (endozoochory: [31]; epizoochory: [32–35]). This indicates that the impact of large herbivores strongly depends on their density and their general behaviour, which includes foraging for specific species, creating disturbances, defecating or wallowing [27]. Taken together, the expected large variation of the impact of large herbivores on understorey plants require well-replicated studies for a comprehensive understanding of the influence of large-herbivore exclusion on forest vegetation [6].

Many studies have shown that understorey vegetation is strongly influenced by different forest features and management intensity [36–38]. An effect of large-herbivore exclusion on understorey vegetation may differ systematically in different forest types, characterised by different forest features and management intensity, either due to the different composition of the understorey vegetation or the influence on the density or identity of large herbivores. For example, the effect of browsing by ungulates on understorey vegetation in low light conditions of a dense forest stand differs from the effect in more open conditions where understorey vegetation cover and species richness is much higher. Even though previous studies suggested that forest features and management possibly influence responses of understorey vegetation to browsing and prevent a final conclusion whether browsing decreases or increases species richness in understorey vegetation (Hester *et al*. 2006), the influence of forest features and management over a large gradient has not been thoroughly tested so far. The two studies that analysed the influence of management on browsing effects on plants differentiated between categories of management only (i.e. clear-cuts and uncut [39]; or clear-cuts, thinning, uncut [40]). It remains important to disentangle how a gradient of different forest features and management intensities influences the possibly context-dependent effect of large herbivores on understorey plants. A large gradient of forest sites with differing forest features, species composition and management intensity needs to be studied.

We experimentally tested the influence of large-herbivore exclusion on forest understorey vegetation, focusing on shrub and herb species, across a wide range of different Central European forests. We excluded large herbivores with fences for seven years on 147 forest sites within the Biodiversity Exploratories program. The forest sites were located in three different regions of Germany (Schwäbische Alb, Hainich-Dün, Schorfheide-Chorin) and included the main forest and management types typical for Central Europe [41]. In particular, we asked i) Do large herbivores reduce the species richness, evenness and vegetation cover of shrubs and herbs? ii) Does the influence of large herbivores on shrub and herb communities depend on certain forest features or management intensity?

## Materials and methods

### Study sites

We conducted this study within the framework of the Biodiversity Exploratories program. The Biodiversity Exploratories program serves as an open research platform to perform long-term and large-scale studies about the relationships between land-use intensity, biodiversity and ecosystem functioning. Our 147 forest study sites are located in three regions of Germany; the Schwäbische Alb (southwestern Germany, 50 sites), the Hainich-Dün (central Germany, 47 sites) and the Schorfheide-Chorin (northeastern Germany, 50 sites). The size of the forest sites is 100 m x 100 m and they are distributed over an area of approximately 30 km x 30 km in each region (S1 Fig). They represent a range of management practices typical for the respective regions, including managed, even-aged forest with conifers replacing the natural vegetation, managed even-aged and managed uneven-aged forests with natural species and formerly managed forests left unmanaged for decades. More information on the sites, the management and the geology, topography and climate of the three regions can be found in [41,42].

### Forest management and structure

To characterize forest management intensity, we used two previously developed indices, the Forest Management Intensity Index ForMI [43] and the Silvicultural Management Index SMI [44]. The ForMI includes the proportion of harvested tree volume, the proportion of tree species that are not part of the natural forest community and the proportion of dead wood that showed signs of saw cuts. The three proportions were then summed, resulting in the ForMI ranging from 0 to 3. The SMI considers an age- and species-specific risk component of stand loss and a density component as a deviation of natural self-thinning. The two components were then summed, resulting in a SMI ranging from 0 to 1.1. The two measures of forest intensity were highly correlated (r = 0.796).

In addition to forest management intensity, we included seven forest features to describe differences among our forest sites. The first forest feature captured variation in soil conditions, as higher nutrient availability might promote regrowth of plants after damage by large herbivores [45]. For this, we used the first axis of a PCA on soil variables, which included the concentrations of nitrogen, carbon, phosphorous, sulphur, soil texture, pH and the content of water and stones (hereafter called PC1 soil). The second feature was the cover of herbs and shrubs, measured independently with vegetation records on 20 m x 20 m plots on each forest site. Higher cover of residential plants may attract more herbivores, thereby potentially increasing browsing levels [46]. Further features were relative conifer cover in percentage of the whole canopy, total canopy cover and the mean diameter at breast height (DBH) of the 50 largest trees measured on each forest site, which are expected to affect understorey vegetation and the density of large herbivores. Details on the measurements of these forest features are in the S1 Material. Variation of the forest features for the different regions are shown with density curves in S2 Fig.

### Large herbivore exclusion experiment

On 150 forest sites, we established a herbivore exclusion experiment in 2008. For this, we fenced an area of 12 m x 12 m with a fence of 190 cm height. The mesh size of the fence increased with increasing height. The mesh size was 5 cm x 15 cm up to 80 cm height, 10 cm x 15 cm from 80–110 cm height and 15 cm x 15 cm above 110 cm. In 2013, we selected and permanently marked two 5 m x 5 m plots on 147 forest sites, one within the fenced area (fenced plots) and one outside the fenced area (unfenced plots). Out of 150 forest sites from the Biodiversity Exploratories program we excluded one forest site due to a severe logging event, making comparisons to other sites impossible and two other forest sites consisting of such a dense thicket that prevented fieldwork. Within forest sites, the fenced and unfenced plots were separated by no more than 5 m and were selected to be similar in terms of tree and shrub layer. As a measure of herbivore pressure, we counted the number of saplings, i.e. trees higher than 20 cm, with a diameter at breast height of less than 7 cm, and recorded the percentage of browsed saplings in the unfenced plots of each site in early spring 2014.

In August 2015 and April 2016, we identified all vascular plant species growing in the fenced and unfenced plots and estimated the cover percentage of each species. With these data we calculated species richness S, exponential of the Shannon index of diversity as exp(H) using the R package ‘vegan’ version 2.4–3 [47] and evenness as evar calculated with the R package ‘codyn’ version 2.0.2 [48]. Additionally, we estimated the total cover percentage of the herb layer (non-woody plants) and shrub layer (woody plants smaller than 5 m). To cover the summer and spring aspects, we recorded the vegetation in summer 2015 and spring 2016, but used the higher cover estimates whenever a species was present in both records.

### Statistical analysis

To analyse the effect of large-herbivore exclusion on understorey vegetation, we calculated the difference (fenced-unfenced) and log response ratio (lnRR) (ln (fenced/unfenced)) of species richness, evenness, exp(H) and total cover separately for herbs and shrubs. We excluded forest sites without any herbs or shrubs on both fenced and unfenced plots. Furthermore, for species that occurred on more than 15 plots, we also calculated the difference and lnRR of their cover. Positive differences and lnRR of larger than zero indicate a positive effect of the exclusion of large herbivores on understorey plants.

To test whether the herbivore pressure, and hence the magnitude of herbivore exclusion, differed among forest sites we calculated a model with regions, forest features and browsing percentage in the unfenced plots as a response variable. Additionally, as we expected large variation in response to herbivore exclusion, we also tested whether the variance of our response variables changed with increasing browsing pressure. Thus, we compared the variance of the response variables among plots below and plots above the median of browsing percentage with an F-test.

We used the differences and lnRRs as response variables in several linear models to test how forest management or forest features influence the effect of large-herbivore exclusion. First, we calculated a linear model with the intercept only, which would indicate an overall significant difference of the vegetation between the fenced and unfenced plot, hence a treatment effect. Second, we calculated linear models containing the region, either of the two forest management indices (SMI or ForMI), and their interaction with region, and browsing percentage as a co-variable. Third, we calculated linear models containing the forest features and the browsing percentage. In that case, we simplified models by minimizing the Akaike information criterion (AIC). We tested all models graphically for normality and heteroscedasticity of the residuals and checked whether correlations between response variables were lower than 0.7 to avoid multicollinearity (S3 Fig).

### Ethics statements

The forest sites are partly owned by private persons and partly owned by the state. Fieldwork permits were issued by the responsible state environmental offices of Baden-Württemberg, Thüringen, and Brandenburg (according to § 72 BbgNatSchG). No rare species were sampled during the fieldwork.

## Results

In the unfenced plots, we found that the percentage of browsed saplings increased from 10% in the Schwäbische Alb and 12% in Hainich-Dün to 28% in Schorfheide-Chorin (S4 Fig). Furthermore, browsing increased in forests with higher cover of herbs (estimate: 0.06) and relative conifer cover (estimate: 1.46). We found traces of browsing on one sapling in each of only two fenced plots in Hainich-Dün. Overall, this indicates that our large-herbivore-exclusion treatment effectively reduced herbivory in our forest sites.

**Fig 1:**
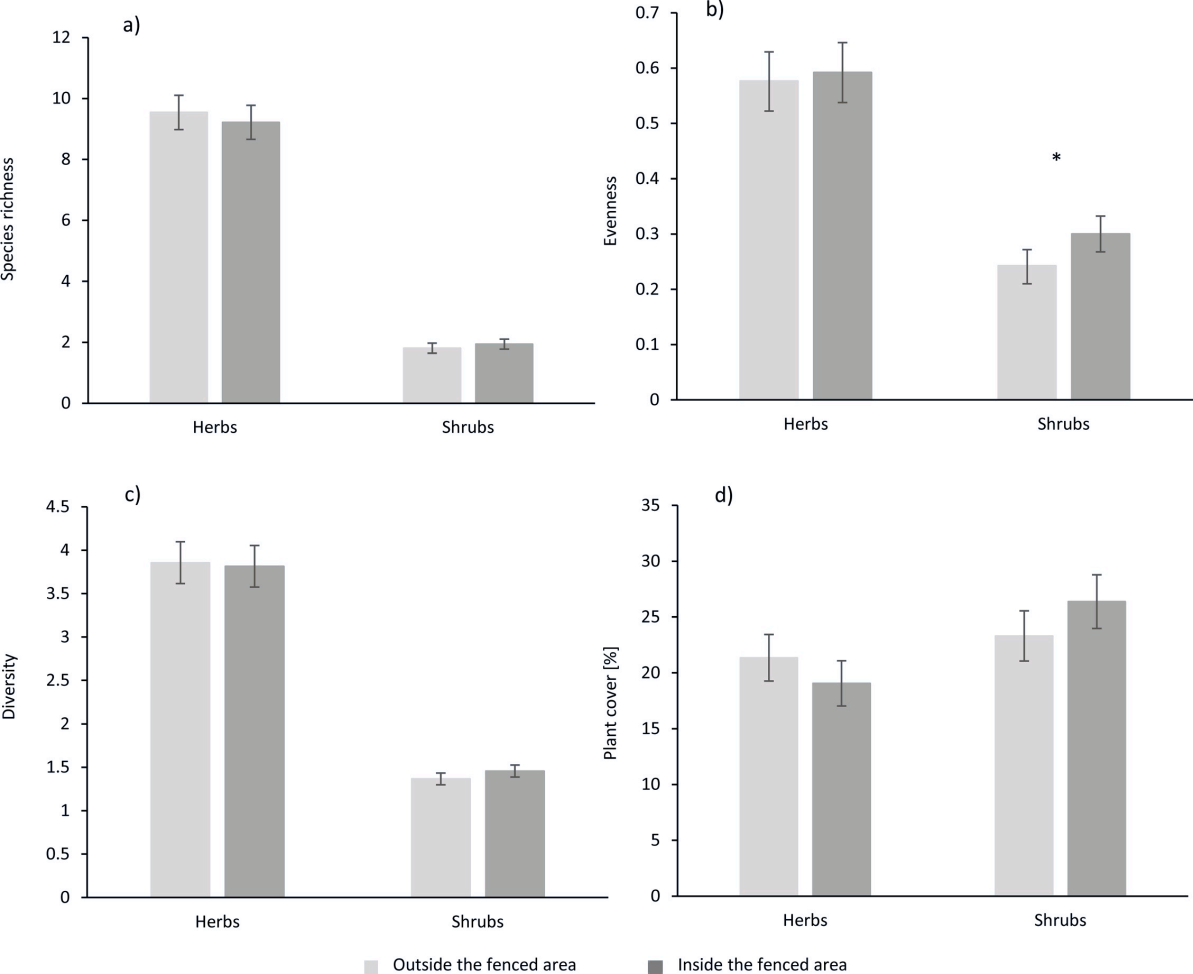
**The effects of herbivore exclusion on (a) species richness, (b) evenness, (c) diversity (exp(H)), (d) plant cover of herbs and shrubs in 147 forest sites of three regions in Germany (means ± standard error).** A star indicates significant difference between the 5 m x 5 m plots outside the fenced area (bright grey) and the 5 m x 5 m plots inside the fenced area (dark grey).

On a relative scale, large-herbivore exclusion increased the total cover and Shannon diversity of shrubs significantly, whereas it did not significantly affect species richness or evenness of shrubs and any measures of herbs (Table 1). On an absolute scale, large-herbivore exclusion increased the cover of shrubs by 13 percentage points, whereas all other measures were not affected significantly (Fig 1, Table 1).

**Table 1:** Influence of forest features on absolute and relative treatment effects.

	Δ (inside—outside the fenced area)							
	Herbs				Shrubs			
	Species richness	Diversity	Evenness	Cover [%]	Species richness	Diversity	Evenness	Cover [%]
Intercept only	-0.336	-0.043	0.024	-1.544	0.129	0.149	0.082	2.974 *
Region	-	-	-	-	-	-	-	*
Mean Schwäbische Alb	-	-	-	-	-	-	-	**0.56**
Mean Hainich-Dün	-	-	-	-	-	-	-	**1.59**
Mean Schorfheide-Chorin	-	-	-	-	-	-	-	**6.68**
PC1soil	-	-	-	-	-	-	-	-
Cover herbs	-	-0.007 *	-0.001.	-	-	-	-	-
Percentage cover conifers	-3.211*	-	-	-13.149 *	-	-	-	-
Mean DBH max 50	-0.075 *	-	-	-	-	-	-	-
Canopy cover	-	-	-	-	-	-	0.610 *	-
Number sapplings	-	-	-	-	-	-	-	-
Browsing percentage	-	-0.007 .	-	-	-	-	-	-
	lnRR (inside /outside the fenced area)							
	Herbs				Shrubs			
	Species richness	Diversity	Evenness	Cover [%]	Species richness	Diversity	Evenness	Cover [%]
Intercept only	-0.014	-0.001	0.055	0.040	0.117	0.062 *	0.109	0.241 *
Region	-	-	-	*	-	-	-	-
Mean Schwäbische Alb	-	-	-	**0.01**	-	-	-	-
Mean Hainich-Dün	-	-	-	**-0.13**	-	-	-	-
Mean Schorfheide-Chorin	-	-	-	**0.28**	-	-	-	-
PC1soil	-	-	-	0.200 .	-	-	-	-0.071 *
Cover herbs	-	-	-	-	-	-	-	-
Percentage cover conifers	-0.352 .	0.241 *	-	-	-	0.241 *	-	-
Mean DBH max 50	-0.009 .	-	-	-	-	-	-	-
Canopy cover	-	-	-	-	-	-	0.596 *	-
Number sapplings	-	-	-	-	-	-	-	-
Browsing percentage	-	-	-	-	-	-	-	-

Results originate from linear models on the change in species richness, diversity, evenness and cover of understorey herbs and shrubs based on the difference (top) and the log-response ratio (lnRR, bottom) of the values inside vs. outside the fenced area in response to region and different forest features. The percentage of browsed tree saplings was also included as a co-variable. Intercept only indicates results from a null-model containing only the intercept. Estimates of the linear models are given after model simplification minimizing AIC. Asterisks indicate significant effects at p < 0.05, the respective estimates are written in bold letters and marginally significant effects at p < 0.1 ˙ are written normally). For significant region effects, we show the mean value of each region.

Among the 152 herb species, 30 species were present on more than 15 forest plots. Three of these were affected by the exclusion of large herbivores. Large-herbivore exclusion increased the abundance of *Arum maculatum* L. (found on 15 plots, lnRR estimate: 0.51, p-value: 0.008) and *Anemone ranunculoides* L. (found on 33 plots, lnRR estimate: 0.81, p-value: 0.09) and decreased the abundance of *Viola reichenbachiana* Boreau (found on 56 plots, lnRR estimate: -0.23, p-value: 0.027).

We also found that the variance of treatment effects was influenced by browsing percentage for some response variables. In forest sites with high browsing percentage the variance of the difference of cover of herbs between fenced and unfenced plots (df: 51, 51; F-value: 2.15) and shrubs (df: 51, 51; F-value: 3.00), and the difference (df: 49, 50; F-value: 4.80) and lnRR (df: 51, 51 F-value: 2.04) of Shannon diversity of herbs was increased.

Both forest management indices did not significantly influence the treatment effects and therefore explained little variation in the effects of large-herbivore exclusion. This indicates that the effects of large-herbivore exclusion were largely independent of management intensity (S1 and S2 Tables).

**Fig 2:**
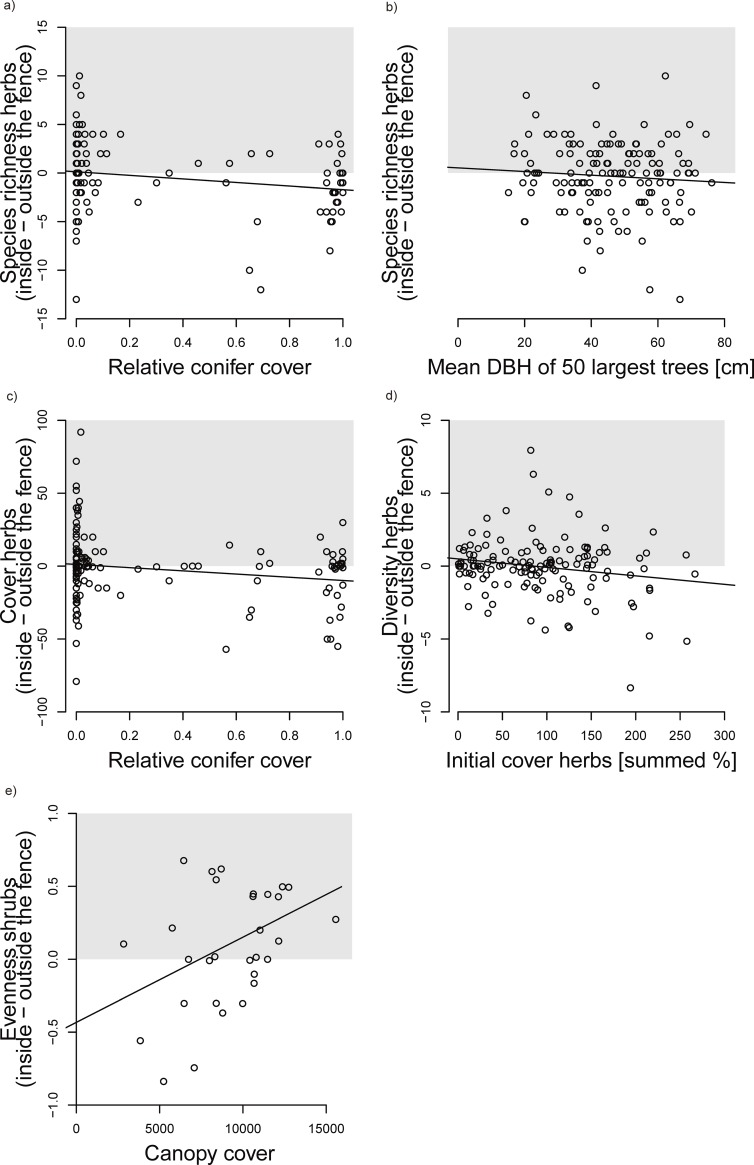
Significant influences of forest features on absolute treatment effects (inside the fence–outside the fence), i.e. the difference. Positive values indicate increased response variables in fenced plots, negative values indicate decreased response values in fenced plots. We see (a) difference of species richness of herbs against relative conifer cover (b) difference of species richness of herbs against mean DBH of the 50 largest trees (c) difference of cover of herbs (all herbs estimated together) against relative conifer cover (d) difference of diversity of herbs (exp(H)) against initial cover of herbs (summed cover of individual species measured independent of treatment plots in spring and summer, resulting in values ranging from 0.1–266) (e) difference of evenness of shrubs against canopy cover.

**Fig 3:**
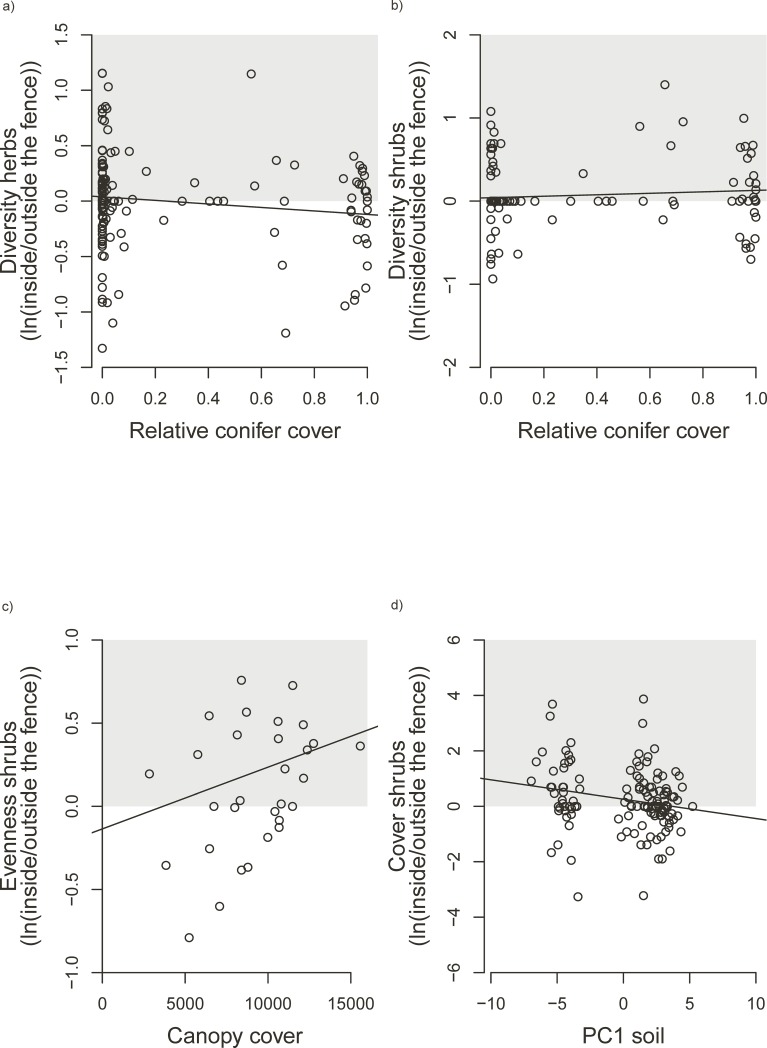
Significant influences of forest features on relative treatment effects (ln(inside the fence/outside the fence)), i.e. lnRR. Positive values indicate increased response variables in fenced plots, negative values indicate decreased response values in fenced plots. We see (a) lnRR diversity (exp(H)) of herbs against percentage cover of conifer (b) lnRR diversity of shrubs against percentage cover of conifers (c) lnRR evenness of shrubs against canopy cover (d) lnRR cover shrubs against PC1 soil (low values indicate clay, alkaline soils, rich in organic nitrogen and high values indicate sandy, acid soils, poor in organic nitrogen).

Some forest features influenced the treatment effects (Table 1, Figs 2 and 3). When considering the difference (inside–outside the fenced area) we found that in forests with high relative conifer cover, large-herbivore exclusion decreased the species richness and cover of herbs, while in forests with low relative conifer cover large-herbivore exclusion increased the species richness and cover of herbs (Fig 2A and 2C). In forests with large mean DBH large-herbivore exclusion decreased the species richness of herbs, while in forests with small mean DBH large-herbivore exclusion increased the species richness of herbs (Fig 2B). In forests with high cover of herbs, large-herbivore exclusion decreased the Shannon diversity of herbs, while in forests with low cover of herbs large-herbivore exclusion increased the Shannon diversity of herbs (Fig 2D). In forests with high canopy cover, large-herbivore exclusion increased the evenness of shrubs (Fig 2E), while in forests with low canopy cover large-herbivore exclusion decreased the evenness of shrubs.

When considering relative values (lnRR (inside/outside the fenced area)) instead of the difference, we found that in forests with high relative conifer cover, large-herbivore exclusion increased the Shannon diversity of herbs (Fig 3A), while in forests with low relative conifer cover large-herbivore exclusion decreased the Shannon diversity of herbs. In forests with high relative conifer cover, large-herbivore exclusion more strongly increased the Shannon diversity of shrubs than in forests with low relative conifer cover (Fig 3B). In forests with high canopy cover, large-herbivore exclusion increased the evenness of shrubs (Fig 3C), while in forests with low canopy cover large-herbivore exclusion decreased the evenness of shrubs. Moreover, we found that in forests with high PC1 soil values (nutrient rich, clayey soils) large-herbivore exclusion increased shrub cover less strongly than in forests with low PC1 soil values (Fig 3C).

## Discussion

Despite the large variability of responses to the exclusion of large herbivores, we found a general increase in Shannon diversity and cover of shrubs after large-herbivore exclusion. The absence of browsing damage allowed for unhindered growth of the shrubs that were already present and increased the Shannon diversity of shrubs. This most likely indicates plant-specific preferences of herbivores, most likely roe deer, which has been shown to have more selective browsing habits than other large herbivores [21,49]. Such selective browsing leads to specific shrub species suffering more than others and those browsing-sensitive shrubs increase in cover when herbivores are excluded. Thus our multi-site study is in agreement with the conclusions of Coté et al. [6] who show in their review that high levels of browsing decreased abundance and complexity in understorey plant communities. Our results are also in line with [50], who found that biodiversity decreased if herbivore changes the dominance structure of the plant communitiy.

In contrast to the shrubs, the exclusion of large herbivores showed no overall effects on species richness, Shannon diversity, evenness and cover of herbs. The absence of any consistent effect of the exclusion of large herbivores on understorey herbs can have several reasons. On the one hand, large variation in our results suggests that the influence of forest features and management intensity on understorey herbs might be stronger than the effect of large herbivores and therefore mask an effect of large-herbivore exclusion on understorey herbs. On the other hand, previous studies have shown that persistent seed banks in forests are scarce [51–53], and that many herbaceous species are dispersal-limited [54] and rely on dispersal by large herbivores [33,55]. The herbaceous plants that we found on our forest sites may display different strategies (resistance or tolerance) to survive under the increasing pressures of large herbivores since the beginning of the twentieth century [5,7,56]. Less defended plants could not be introduced in the fenced plots or did not establish during the seven years of large herbivore exclusion. Alternatively, a negative effect of large herbivores on herbaceous plants via browsing may have been compensated by a positive effect of seed dispersal by large herbivores in the unfenced plots [57] or a negative effect of increased shrub cover on light availability for herbs in the fenced plots [24,58]. Further, the effect of non-migratory, large herbivores on forest vegetation might be strongest in winter when food is limited, which could at least partly explain the lack of an effect on understorey herbs. Instead, seven years of herbivore exclusion may have been too short for inducing major shifts in the understorey vegetation as previous studies also suggested that overbrowsing by large herbivores can push plant communities into an alternative stable state, from which recovery is expected to be slow [19,59,60]. The increase in shrub cover together with the lack of seed dispersal and a generally slow recovery of the understorey plant community after large-herbivore exclusion could explain our findings.

We found little evidence that different forest features or management intensity influenced the effects of large-herbivore exclusion on the understorey vegetation. For example, relative conifer cover, the forest feature that most often influenced treatment effects, correlated positively with browsing percentage, initial shrub cover and a more diverse understorey. Thus, the influence of relative conifer cover on the treatment effects could partly be caused by an underlying increase of herbivore pressure, which increased with relative conifer cover or a stronger increase of the Shannon diversity of shrubs and herbs in fenced plots, because competitive species, that are undefended, could increase their cover more in more diverse understoreys. Many previous studies reporting that forest features and management have strong influences on understorey vegetation have not considered effects of large herbivores [36,38,61,62]. Our study suggests that the effects of large herbivores on understorey vegetation, especially of herbs, is indeed relatively small compared with the effects of forest features and management intensity and can be dependent on them.

Consequences of excluding large herbivores strongly depend on the actual density of large herbivores and their general behaviour at the sites, such as foraging for specific species, creating disturbances, defecating or wallowing [6]. All these behaviours may affect plant communities, but are highly context-specific. We therefore expected large variation in our treatment effect, which can make it difficult to find significant effects, even if sample size is large [27]. Part of the large variation found in our data could also be caused by small-scale differences of the fenced and unfenced areas, which could not be quantified by our explanatory variables, which were partly measured on a larger scale. Nevertheless, our results support that variation in treatment effects was higher at sites with higher browsing percentage.

## Conclusion

We advocate the implementation of long-term studies over several decades to analyse the potentially very slow recovery of understorey plant communities after large-herbivore exclusion. We also stress the need of large-scale studies to account for the large variation in the response of understorey plant communities after exclusion of large herbivores. Despite large variation in the response of understorey plants to large-herbivore exclusion, we found some consistent changes in shrub cover and Shannon diversity, most likely caused by selective browsing of herbivores. However, we did not find changes of herbaceous vegetation seven years after large-herbivore exclusion, indicating that, compared with other forest features and management intensity, browsing is not strongly influencing understorey herbs. Lastly, we suggest that browsing should generally be considered when studying understorey shrubs. When studying understorey herbs browsing seemed to be generally of lesser importance, however it should still be considered in interaction with other forest feature and management.

## Supporting information

S1 Material Details on the measurements of the forest features. (DOCX)

S1 Fig Maps showing the location of the three study regions Schwäbische Alb, Hainich-Dün and Schorfheide-Chorin within Germany and the distribution of the 147 forest sites within the regions. The three main forest types conifer managed, broadleaf managed and broadleaf unmanaged are marked with different colours.
(DOCX)

S2 Fig Density curves of the eight different forest features showing the range and variation within and between the three different study regions. The three different study regions are indicated with different colours. Schwäbische Alb in yellow, Hainich-Dün in red and Schorfheide-Chorin in blue.
(DOCX)

S3 Fig Spearman correlation of all explanatory variables included in our study and the cover of herbs and shrubs and species richness of herbs and shrubs on the unfenced 5 m x 5 m plots from 147 forest sites. Red squares indicate negative correlations and blue squares indicate positive correlations. The more intense the colour the stronger the correlation.
(DOCX)

S4 Fig Browsing intensity in 147 forest sites. Browsing intensity is indicated as the percentage of browsed saplings on a 5 m x 5 m plot in three regions in Germany (mean ± standard error).
(DOCX)

S1 Table Influence of ForMI (forest management intensity index) on absolute and relative treatment effects. Results originate from linear models on the change in species richness, diversity, evenness and cover of understorey herbs and shrubs based on the difference (top) and the log-response ratio (lnRR, bottom) of the values inside vs. outside the fenced area in response to region and ForMI. On the left hand side, the response variables are from the herb layer and on the right hand side, the response variables are from the shrub layer. Estimates of the linear models are given for significant results only. Stars indicate the p-value (*** p <0.001, ** 0.001 < p < 0.01, * 0.01 < p < 0.05, 0.05 < p < 0.1 ˙). For significant region effects, we show the mean value of each region.
(DOCX)

S2 Table Influence of SMI (silvicultural management intensity index) on absolute and relative treatment effects. Results originate from linear models on the change in species richness, diversity, evenness and cover of understorey herbs and shrubs based on the difference (top) and the log-response ratio (lnRR, bottom) of the values inside vs. outside the fenced area in response to region and SMI On the left hand side, the response variables are from the herb layer and on the right hand side, the response variables are from the shrub layer. Estimates of the linear models are given for significant results only. Asterisk are given to indicate the p-value (*** p <0.001, ** 0.001 < p < 0.01, * 0.01 < p < 0.05, 0.05 < p < 0.1 ˙). For significant region effects, we show the mean value of each region.
(DOCX)
