# Supplementary material for: Exclusion of large herbivores affects understorey shrub vegetation more than herb vegetation across 147 forest sites in three German regions
Source: PLoS One. 2019 Jul 10;14(7):e0218741. doi: 10.1371/journal.pone.0218741 (PMC6619654; doi:10.1371/journal.pone.0218741)
Supplement: S1 Material — (DOCX) [file pone.0218741.s001.docx]

**S1 Material:** Details on the measurements of the forest features.

**PC1 soil**: During a soil sampling campaign in 2011 that took place on all 150 forest sites we measured Nitrogen, Carbon, Phosphorus, Sulphur, pH, soil texture, water content, dry matter and stones.

We measured *Nitrogen and Carbon* concentrations in organic and mineral layers. We dried organic horizons at 60°C. From mineral soils we collected the top 10 cm and air-dried the samples. For the analyses, we then cut and ground roughly 20 mg from organic soils and 250 mg from mineral soils. We treated the samples with dry combustion at 1100 °C and determined CO_2_ and N_2_ with a Thermal Conductivity Detector. To determine inorganic carbon we removed organic carbon at 450 °C for 16 h.

We measured *Phosphorus and Sulphur* concentrations in the top 10 cm of mineral soils. We air-dried the samples, sieved them to < 2 mm, grounded them and took a sample of 1.5 g for the analysis. We extracted Phosphor and Sulphur with aqua regia by adding 3.5 ml HNO_3_ and 10.5 ml HCL. Then we boiled the samples at 96 °C for 3h, then diluted and filtered them.

We measured *pH* in the top 10 cm of mineral soils in a 1:2.5 soil:0.01 M CaCl_2_ suspension.

To analyse the *soil texture* we determined the percentage of sand (2-0.063 mm), silt (0.063-0.002 mm) and clay (< 0.002 mm) in the soil samples. Additionally, we subdivided sand and silt into coarse, medium and fine sand respectively silt. The methods used were sieving and sedimentation (DIN-ISO 11277).

To measure *water content and dry matter* we dried the soil samples until constant weight at 105 °C, weighted the moist and dry soil and calculated the percentage of water per dry soil.

With a shredding machine (organic layers), a sieving machine (loamy and clayey mineral soil) and a 2 mm sieve (sandy soil) we separated *stones* and weighted them.

With all soil variables we did a principal component analysis and used the first axis (64.9 % of variance) corresponding to total nitrogen content, variability in soil texture and pH as a covariate in our linear models. A positive value indicates clay soils, rich in organic nitrogen and with an alkaline pH. While a negative value indicates sandy soils, poor in organic nitrogen and with an acid pH.

Additionally to the vegetation records on the 5 m x 5m fenced and unfenced plots we also recorded the understorey vegetation (**cover of shrubs and herbs**) in spring and summer 2015 on a 20 m x 20 m area on all 150 forest sites. If a species occurred in both, spring and summer, vegetation records, we kept the higher cover value. Finally, we summed the cover estimate of each herb, respectively shrub species, which resulted in values from 0.1-266%. From 2008 until 2011, we surveyed, on all 150 100 m x 100 m forest sites, all trees that had a diameter at breast height larger than 7 cm. For each tree, we measured canopy cover as well as DBH, from which we could deduce total **canopy cover**, **mean diameter of the 50 largest trees** and the **percentage cover of conifer** species (i.e. relative conifer cover).

On the 5 m x 5 m fenced and unfenced plots we counted the number of all tree species with a DBH smaller than 7 cm. We refer to this measurement as the **number of saplings**. Additionally, we noted if the saplings showed damage caused by game animals, calculated the percentage of damaged saplings from the total number of saplings, and referred to this as **browsing percentage**.
