# Supplementary material for: Exclusion of large herbivores affects understorey shrub vegetation more than herb vegetation across 147 forest sites in three German regions
Source: PLoS One. 2019 Jul 10;14(7):e0218741. doi: 10.1371/journal.pone.0218741 (PMC6619654; doi:10.1371/journal.pone.0218741)
Supplement: S1 Fig — The three main forest types conifer managed, broadleaf managed and broadleaf unmanaged are marked with different colours. (DOCX) [file pone.0218741.s002.docx]

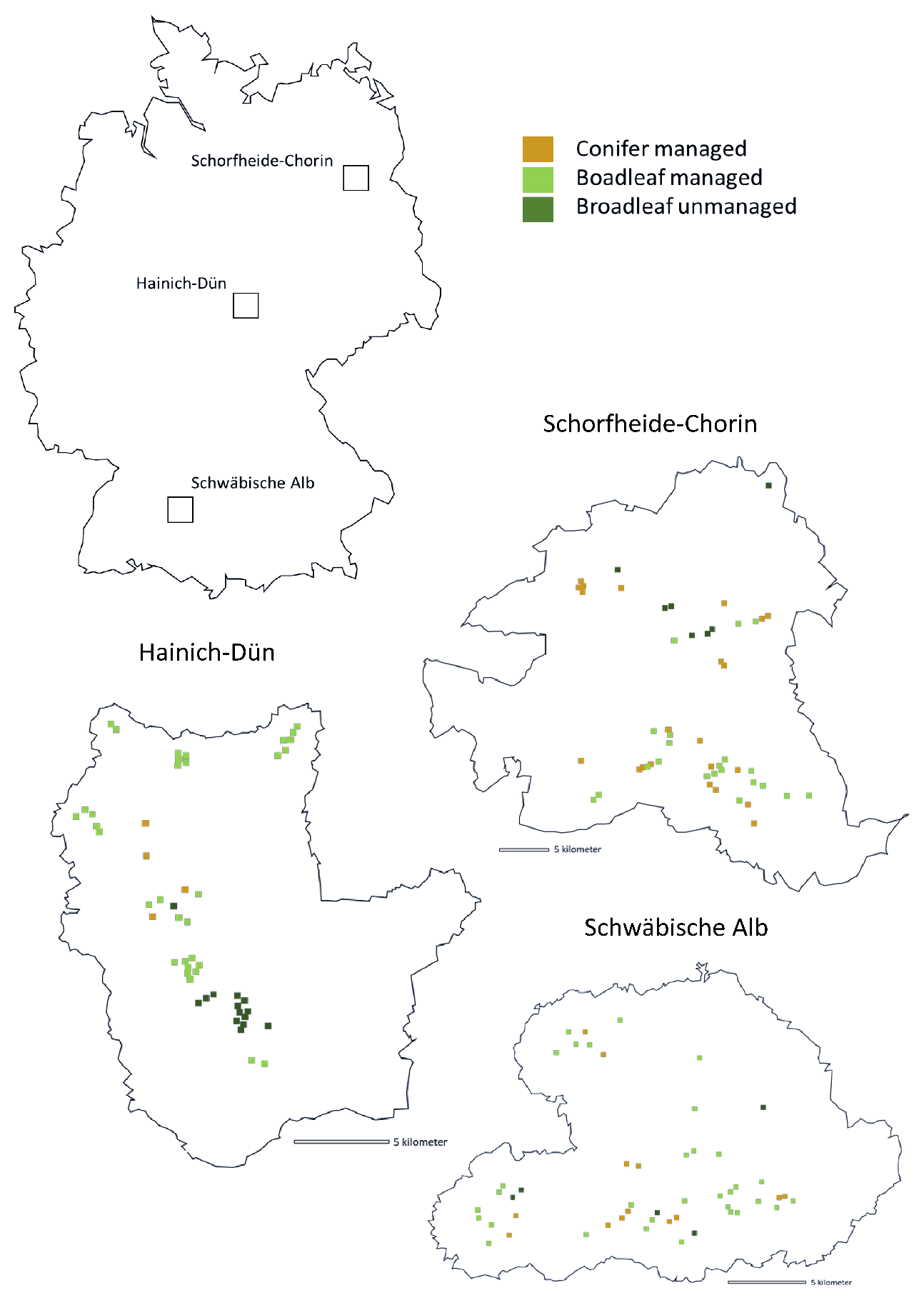


**S1 Figure: Maps showing the location of the three study regions Schwäbische Alb, Hainich-Dün and Schorfheide-Chorin within Germany and the distribution of the 147 forest sites within the regions.** The three main forest types ‘conifer managed’, ‘broadleaf managed’ and ‘broadleaf unmanaged’ are marked with different colours.
