# Supplementary material for: Exclusion of large herbivores affects understorey shrub vegetation more than herb vegetation across 147 forest sites in three German regions
Source: PLoS One. 2019 Jul 10;14(7):e0218741. doi: 10.1371/journal.pone.0218741 (PMC6619654; doi:10.1371/journal.pone.0218741)

**S2 Figure: Density curves of the eight different forest features showing the range and variation within and between the three different study regions.** The three different study regions are indicated with different colours. Schwäbische Alb in yellow, Hainich-Dün in red and Schorfheide-Chorin in blue.
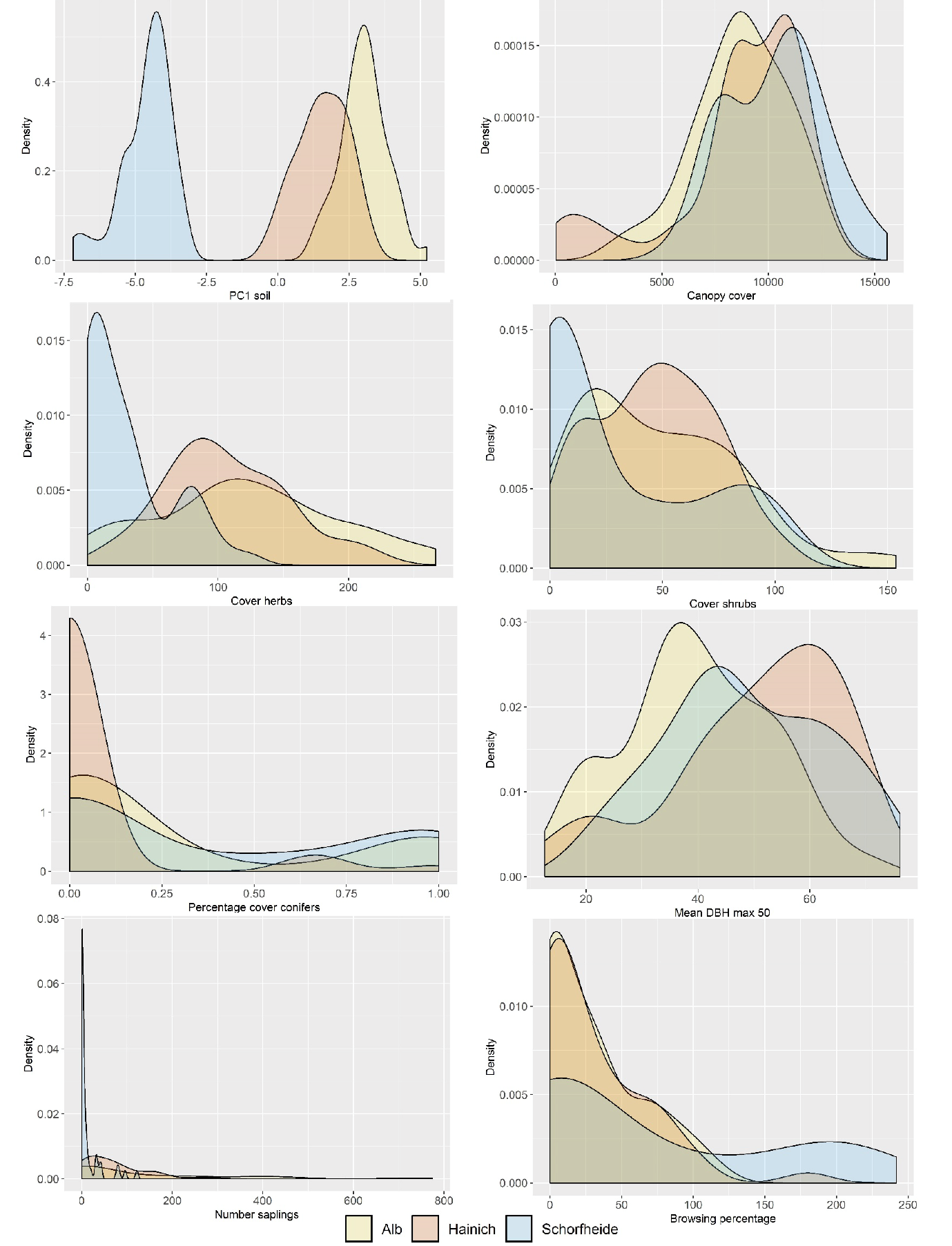

Supplement: S2 Fig — The three different study regions are indicated with different colours. Schwäbische Alb in yellow, Hainich-Dün in red and Schorfheide-Chorin in blue. (DOCX) [file pone.0218741.s003.docx]
