# Supplementary material for: Exclusion of large herbivores affects understorey shrub vegetation more than herb vegetation across 147 forest sites in three German regions
Source: PLoS One. 2019 Jul 10;14(7):e0218741. doi: 10.1371/journal.pone.0218741 (PMC6619654; doi:10.1371/journal.pone.0218741)
Supplement: S3 Fig — Red squares indicate negative correlations and blue squares indicate positive correlations. The more intense the colour the stronger the correlation. (DOCX) [file pone.0218741.s004.docx]

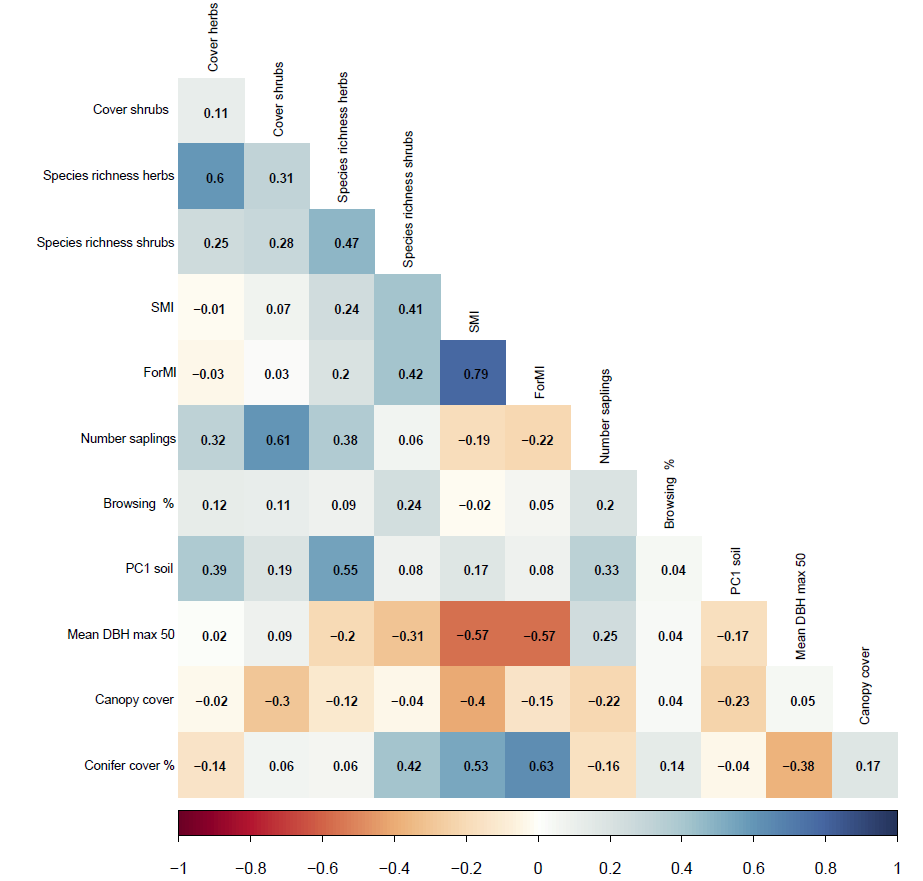


**S3 Figure: Spearman correlation of all explanatory variables included in our study and the cover of herbs and shrubs and species richness of herbs and shrubs on the unfenced 5 m x 5 m plots from 147 forest sites.** Red squares indicate negative correlations and blue squares indicate positive correlations. The more intense the colour the stronger the correlation.
