# Supplementary material for: Exclusion of large herbivores affects understorey shrub vegetation more than herb vegetation across 147 forest sites in three German regions
Source: PLoS One. 2019 Jul 10;14(7):e0218741. doi: 10.1371/journal.pone.0218741 (PMC6619654; doi:10.1371/journal.pone.0218741)
Supplement: S4 Fig — Browsing intensity is indicated as the percentage of browsed saplings on a 5 m x 5 m plot in three regions in Germany (mean ± standard error). (DOCX) [file pone.0218741.s005.docx]

Browsing percentage


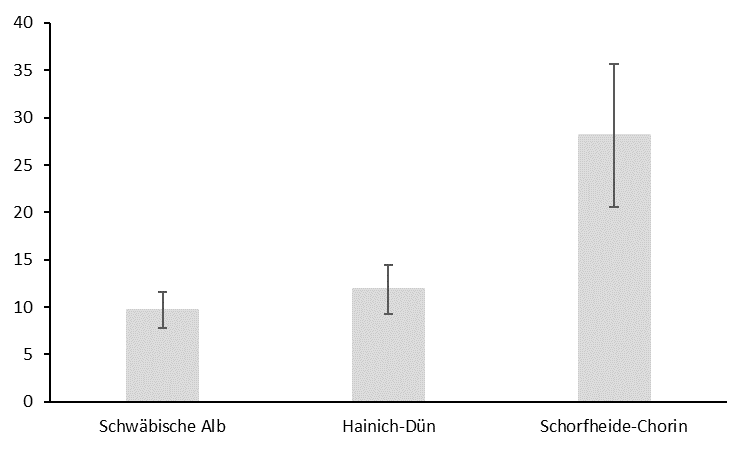


**S4 Figure: Browsing intensity in 147 forest sites.** Browsing intensity is indicated as the percentage of browsed saplings on a 5 m x 5 m plot in three regions in Germany (mean ± standard error).
