# Supplementary material for: Exclusion of large herbivores affects understorey shrub vegetation more than herb vegetation across 147 forest sites in three German regions
Source: PLoS One. 2019 Jul 10;14(7):e0218741. doi: 10.1371/journal.pone.0218741 (PMC6619654; doi:10.1371/journal.pone.0218741)
Supplement: S1 Table — Results originate from linear models on the change in species richness, diversity, evenness and cover of understorey herbs and shrubs based on the difference (top) and the log-response ratio (lnRR, bottom) of the values inside vs. outside the fenced area in response to region and ForMI. On the left hand side, the response variables are from the herb layer and on the right hand side, the response variables are from the shrub layer. Estimates of the linear models are given for significant results only. Stars indicate the p-value (*** p <0.001, ** 0.001 < p < 0.01, * 0.01 < p < 0.05, 0.05 < p < 0.1 ˙). For significant region effects, we show the mean value of each region. (DOCX) [file pone.0218741.s006.docx]

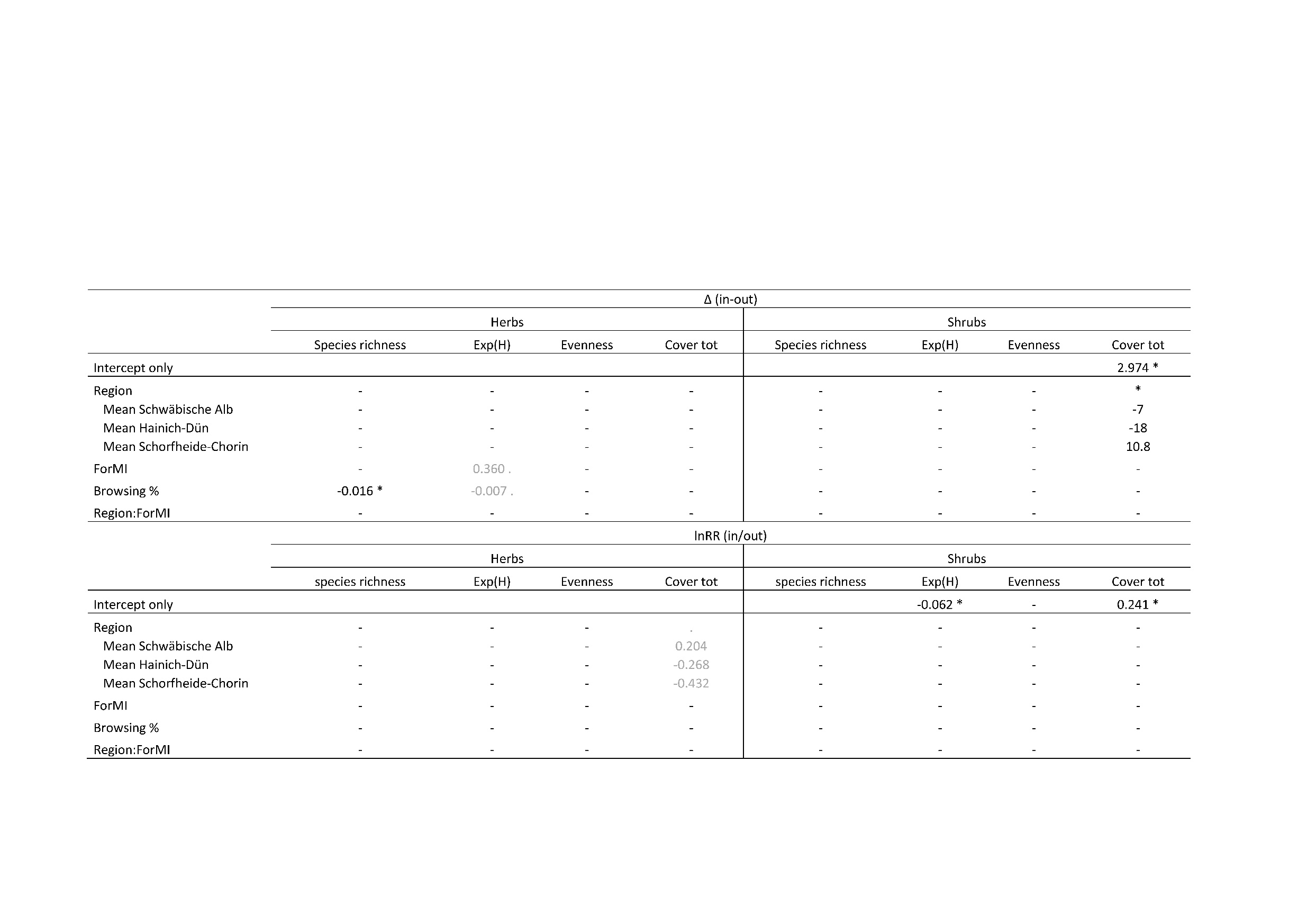
**S1 Table**: **Influence of ForMI (forest management intensity index) on absolute and relative treatment effects**. Results originate from linear models on the change in species richness, diversity, evenness and cover of understorey herbs and shrubs based on the difference (top) and the log-response ratio (lnRR, bottom) of the values inside vs. outside the fenced area in response to region and ForMI. On the left hand side, the response variables are from the herb layer and on the right hand side, the response variables are from the shrub layer. Estimates of the linear models are given for significant results only. Stars indicate the p-value (*** p <0.001, ** 0.001 < p < 0.01, * 0.01 < p < 0.05, 0.05 < p < 0.1 ˙). For significant region effects, we show the mean value of each region.
